# sST2: A Bridge Between Sirt1/p53/p21 Signal-Induced Senescence and TGF-β1/Smad2/3 Regulation of Cardiac Fibrosis in Mouse Viral Myocarditis

**DOI:** 10.1007/s10753-023-01809-2

**Published:** 2023-04-10

**Authors:** Jiajia Tan, Jing Wei, Hongxiang Lu

**Affiliations:** 1 International Genome Center, Jiangsu University, Zhenjiang, China; 2 Department of Laboratory Medicine, Jiangning Hospital Affiliated to Nanjing Medical University, Nanjing, 211100 China; 3 Department of Laboratory Medicine, Nanjing First Hospital, Nanjing Medical University, Nanjing, 210006 China

**Keywords:** sST2, cardiac fibroblasts, viral myocarditis, cellular senescence, cardiac fibrosis.

## Abstract

Soluble interleukin 1 receptor-like 1 (sST2) is a novel predictor of poor outcomes, which is involved in inflammatory response and fibrosis of myocarditis. Cellular senescence is a state of irreversible cell cycle arrest. Studies have shown that senescence of myofibroblasts can limit or reduce cardiac fibrosis. However, the molecular mechanism of sST2 regulating cellular senescence is still unclear. Here, we investigate the role of sST2 on cellular senescence in cardiac fibrosis. Our results found that sST2 was upregulated in coxsackievirus group B type 3 (CVB3)-induced viral myocarditis (VMC), which correlated with the expression of senescence markers. *In vitro*, sST2 activated TGFβ signaling through the phosphorylation of the SMAD complex to induce mouse cardiac fibroblast (MCF) activation and inhibit cellular senescence by the Sirt1/p53/p21 signaling pathway. *In vivo*, anti-ST2 mAb attenuated CVB3-induced cardiac fibrosis. Our findings elucidate a crucial mechanism underlying through which sST2 inhibits cellular senescence and regulates MCF activation, providing a potential treatment strategy for cardiac fibrosis.

## INTRODUCTION

Viral myocarditis (VMC) is the leading cause of sudden cardiac death in children and adolescents [1]. Many viruses, including enteroviruses, adenoviruses, and human herpes virus 6, are associated with VMC [2, 3]. Among these, coxsackievirus group B type 3 (CVB3), an enterovirus of the Picornaviridae family, is known as the main etiological agent in VMC. In the early stages of CVB3-induced VMC, immune cells infiltrate the infected tissues which lead to infiltration of a large number of damage-related molecular patterns. Among them, sST2 is a research hotspot and is considered to be a valuable predictor of cardiovascular disease. In the later stages, fibrosis occurs in the heart due to the activation of cardiac fibroblasts, leading to increased production of extracellular matrix (ECM) [4–6].

The protein ST2 is encoded by the interleukin 1 receptor-like 1 (*IL1RL1*) gene, which belongs to the IL-1 receptor family [7]. Alternative splicing of *IL1RL1* results in multiple transcript variants, including the membrane-bound ST2L receptor for IL-33 and a soluble inhibitory decoy receptor, sST2. When the heart is stressed or stimulated, mouse cardiac fibroblasts (MCFs) produce large amounts of sST2 [8]. The imbalance of sST2 content greatly accelerates the progression of cardiac fibrosis and causes serious cardiomyopathy, but most of the studies have shown that sST2 acts only as a decoy receptor, which prevents IL-33/ST2L signaling [9]. Indeed, sST2 can work independently of IL-33 signal [10]. Recent studies have shown that recombinant sST2 promotes mitochondrial fusion in human cardiac fibroblasts, increasing oxidative stress and the secretion of inflammatory cytokines [11]. High levels of sST2 have been found to be associated with poor prognosis of cardiac fibrosis and cardiomyopathy, which can be used as an independent predictor of heart disease [12–14]. However, there are various mechanisms that cause fibrosis, and the mechanism of myocardial fibrosis caused by sST2 remains unclear.

Numerous studies have shown that cellular senescence and fibrosis are closely related. Cellular senescence is a dynamic condition that can lead to irreversible cell cycle arrest [15, 16]. Although most of researches have shown that senescence contributes to the development of fibrosis, several recent studies have also found that senescence can reduce myocardial, hepatic, and idiopathic pulmonary fibrosis [17, 18]. miR-486 increases the expression of p21 and decreases the expression of fibrotic effector genes in the heart after myocardial infarction [19]. TGFβ1 stimulation in idiopathic fibrosis significantly increases the activity of SA-β-gal and the levels of senescence-related proteins p21 and p53 in lung fibroblasts. Mannose lectin limits the progression of liver fibrosis by promoting hepatic stellate cellular senescence [17]. Therefore, there is an inextricable, though somewhat unpredictable, relationship between cellular senescence and fibrosis.

At present, to our knowledge, few studies have described a relationship between sST2 and cardiac fibrosis by means of inhibiting cellular senescence. The aim of this study is to provide a mechanistic assessment of sST2 on the activation of MCFs in VMC from the point of view of cellular senescence.

## MATERIALS AND METHODS

### Mice

Male BALB/c (6–8 weeks) mice were obtained from Kavins Laboratory Animal Company (Changzhou, China). All animal experiments were performed in accordance with the guidelines for the Care and Use of Laboratory Animals (Ministry of Health, China, 1998).

### CVB3 Infection and Anti-ST2 mAb Treatment

CVB3 virus (Nancy strain) was maintained by passage through Hela cells (ATCC number: CCL-2). Mice were infected with CVB3 via intraperitoneal (*i.p.*) injections at the dose of 10^4^ 50% tissue culture infectious dose (TCID50) of CVB3. Anti-ST2 mAb (R&D systems, MAB10041) were given to VMC mice by intraperitoneal (*i.p.*) injection the day before and the day after the infection (20 μg/mouse). Seven days or fifteen days later, the hearts and serum were collected for the experiment.

### Cell Culture

MCF were purchased from BNCC (Beijing, China) and were cultured with DMEM medium (Gibco) containing 10% FBS in a 5% CO^2^ incubator. The 3–6 passages of cells were used for experiments. Cells were stimulated with sST2 (50 nM, R&D Systems) for 12 h or 24 h. The 100 μM H_2_O_2_ were added for 2 h prior to the stimulation with sST2.

### Quantitative RT-PCR

Using an RNA extraction kit (Invitrogen), total RNA was extracted from cells and tissues. Purified RNA was reverse transcribed into cDNA, then amplified by SYBR-Green master mix kit. Real-time PCR primer sequences were as follows: for mouse p53, 5′-GCGTAAACGCTTCGAGATGTT-3′ (forward) and 5′-TTTTTATGGCGGGAAGTAGACTG-3′ (reverse); mouse Sirt1, 5′-ATGACGCTGTGGCAGATTGTT-3′ (forward) and 5′-CCGCAAGGCGAGCATAGAT-3′ (reverse); mouse p21, 5′-GTGATTGCGATGCGCTCATG-3′ (forward) and 5′-TCTCTTGCAGAAGACCAATC-3′ (reverse); mouse p16, 5′-AGGGCCGTGTGCATGACGTG -3′ (forward) and 5′-GCACCGGGCGGGAGAAGGTA-3′ (reverse); Mouse GAPDH, 5′-AGGTCGGTGTGAACGGATTTG-3′ (forward) and 5′-GGGGTCGTTGATGGCAACA-3′ (reverse). Relative levels of gene expressions were determined by the relative standard curve method and normalized to GAPDH and β-actin.

### Western Blot

RIPA buffer was used for preparing whole cell lysates. Protein was separated by SDS-PAGE and transferred to PVDF membranes (Millipore). In order to block the membranes, 1% BSA was added and the membranes were washed three times with TBS-0.1% Tween 20 (TBST). The washed membranes were incubated primary antibodies at 4˚C overnight. The following primary antibodies were used: Collagen-I (Immunoway, YT6135, 1:1000), Collagen-III (Proteintech, 22,734–1-AP, 1:500), α-SMA (Proteintech, 14,395–1-AP, 1:3000), p16 (Immunoway, YT5664, 1:1000), p21 (Immunoway, YT3497, 1:1000), p53 (Baijia, IMB1162, 1:1000), Sirt1 (Cohesion, CQA2569, 1:1000), sST2 (Proteintech, 60,112–1-lg, 1:5000), TGF-β1 (Cohesion, CPA2154, 1:1000), and p-Smad2/3 (Bioworld, AP0326, 1:1000). Afterwards, the secondary antibody (1:8000; Abcam) was incubated at room temperature for 1 h developed using an ECL chemiluminescence kit. Finally, the antigen–antibody reactions were visualized by chemiluminescence (ECL) kit, and the intensity of protein bands was quantified by using ImageJ software.

### ELISA

Soluble sST2 levels were quantified in serum according to the manufacturer’s instructions. The results were normalized to the control condition. Data were expressed as a fold change relative to the control conditions.

### CCK-8 Assay

MCF were seeded into 96-well plates at 5000 cells per well 24 h before treatment, and three replicate experiments were performed in each group of cells. At the indicated time points, 10 μL CCK8 solution was added to each well and incubated at 37 °C with 5% CO^2^ for 3 h. Finally, the absorbance was detected using a microplate reader (Thermo Fisher Scientific, Waltham, MA, USA) at 450 nm. The higher the absorbance in OD450, the more active the cell.

### Examination of Myocardial Markers

The levels of lactate dehydrogenase (LDH), creatine kinase-MB (CK-MB), and glutamic oxalacetic transaminase (AST) in serum were measured using the available commercial kits (JianCheng, Nanjing, China), according to the manufacturer’s instructions.

### Senescence Detection

A β-galactosidase Staining Kit (Solarbio, Beijing, China) was employed to detect the senescence of MCFs after the cells were treated with sST2 and H_2_O_2_. The short answer is that cells were seeded in a twelve-well plate. The medium was removed before the experiment and washed with phosphate-buffered saline (PBS; D8537, Sigma) once. Then, 1 mL 4% paraformaldehyde was added at room temperature. After 15 min, the 4% paraformaldehyde was removed and the cells were washed with PBS for three times (3 min/time). Subsequently, 1 mL of β-galactosidase staining working solution was added and incubated overnight at 37 °C. Observe cells under microscope (CKX53, Olympus) and take photos. Finally, the senescence of the cells was examined by using ImageJ software.

### Histological Examination of the Heart

The heart tissues were fixed in 4% polyformaldehyde and embedded in paraffin. Then sections were stained with hematoxylin–eosin (H&E). H&E staining was used to analyze the level of inflammation in the heart.

### Immunofluorescence Staining

Hearts were resected and fixed in 4% paraformaldehyde for 24 h and dehydrated with 30% sucrose for 2 h. Then, the specimens were processed into frozen sections. Sections of the heart were permeabilized in 0.5% Triton X-100 for 20 min. Then, 5% BSA was dripped into the tissue on the section and wait for 1 h. Afterwards, the sections were incubated with anti-mouse p21 and anti-mouse α-SMA antibodies at 4 ℃ overnight. After being washed, fluorescent secondary antibody were applied. Finally, nuclei were identified with DAPI (Thermo Fisher Scientific) and representative figures were taken by a fluorescence microscope.

### Statistical Analysis

All data were analyzed using GraphPad Prism 8.0 (GraphPad Software, Inc.). In ImageJ, grayscale scans were performed on Western blot analysis results. The data were expressed as the mean ± standard deviation. A *t*-test was used to compare the data between two groups, and the differences between multiple groups were analyzed via a one-way analysis of variance. A *p* value of 0.05 or less was considered statistically significant.

## RESULTS

### sST2 Increases and Promotes Inflammatory Damage During Viral Myocarditis

**Fig. 1:**
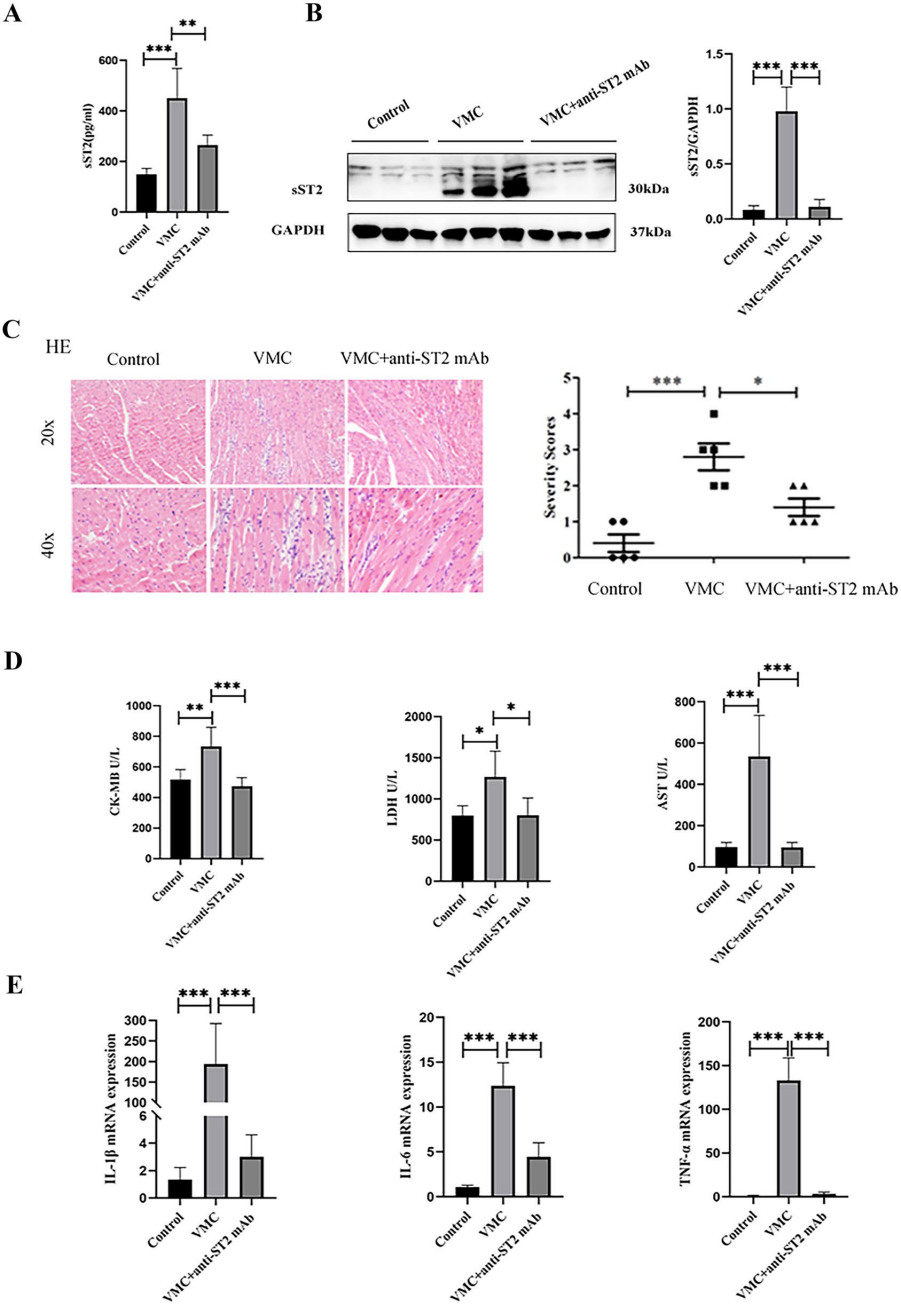
sST2 increased and promoted inflammatory damage in the viral myocarditis. **A** The generation of sST2 was determined using ELISA. **B** Western blot analyses of protein expression of sST2. **C** H&E stain was used to evaluate the degree and grade of cardiac inflammation. **D** LDH, CK-MB, and AST levels in the serum were determined using a fully automatic biochemical analyzer. **E** RT-PCR analyses of the mRNA levels of IL-1β, IL-6, and TNF-α. Data were represented as the mean ± S.D. Compared with the control group, *p < 0.5; **p < 0.01; ***p < 0.001.

To investigate the relationship between viral myocarditis and sST2, mice were divided into three groups: control, VMC, and VMC + anti-ST2 mAb. First, sST2 content in the serum and heart tissue of mice was detected. We observed that VMC increased the expression and secretion of sST2, while anti-ST2 mAb injection effectively reduced both (Fig. 1A, B). Since VMC can cause cardiac inflammatory cell infiltration, fibrosis, and cardiac injury, we investigated whether sST2 was associated with these pathological phenomena [20]. By hematoxylin and eosin staining, we found that anti-ST2 mAb could reduce the infiltration of cardiac inflammatory cells, as well as creatine kinase-MB, lactate dehydrogenase, and aspartate aminotransferase contents in the sera of VMC mice (Fig. 1C, D). Consistent with the above results, anti-ST2 mAb decreased the transcriptional abundance of *Il1b*, *Il6*, and *Tnfa*, as measured by RT-PCR (Fig. 1E). Together, these results suggest that viral myocarditis increases the protein expression of sST2 and that sST2 in turn promotes cardiac inflammation.

### sST2 Promotes the Activation of MCF and Inhibits Its Senescence *In Vitro*

**Fig. 2:**
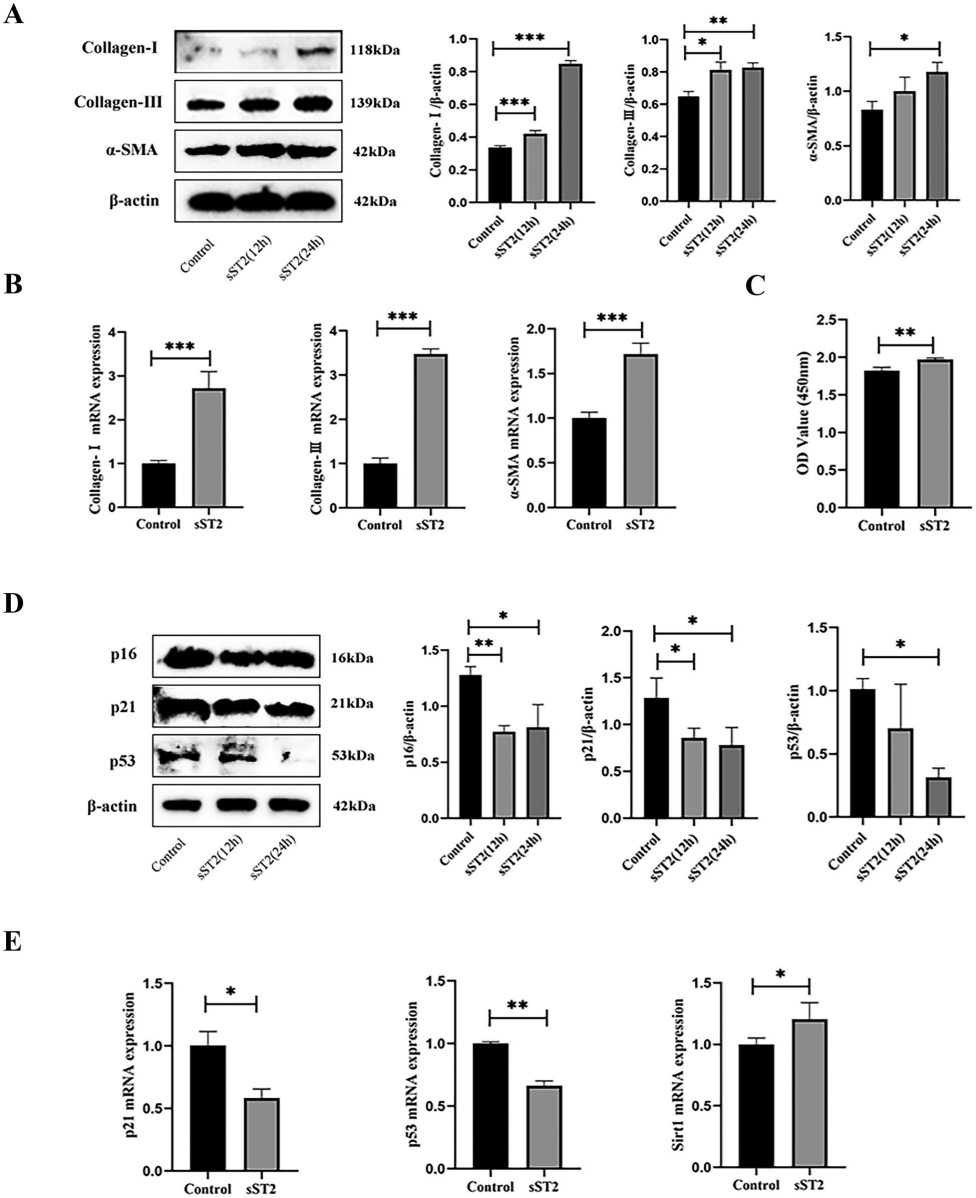
sST2 promoted the activation of MCF and inhibits its senescence *in vitro*. **A** Western blot analyses of protein expression of Collagen-I, Collagen-III, and α-SMA. **B** RT-PCR analyses of the mRNA levels of Collagen-I, Collagen-III, and α-SMA. **C** CCK8 assay was used to detect proliferation ability of MCFs. **D** Western Blot analyses of protein expression of p16, p21, and p53. **E** RT-PCR analyses of the mRNA levels of p21, p53, and Sirt1. Data were represented as the mean ± S.D. Compared with the control group, *p < 0.5; **p < 0.01; ***p < 0.001.

Having found that sST2 can promote cardiac fibrosis *in vivo*, we further explored the relationship between sST2 and MCFs *in vitro*. Western blot analysis was used to detect whether sST2 could promote the expression of fibrosis-related proteins. The results showed that sST2 upregulated the expression of the fibrosis-related proteins Collagen-I, Collagen-III, and αSMA in MCFs (Fig. 2A). In addition, we also confirmed that sST2 upregulated the expression of the fibrosis-related genes Collagen-I, Collagen-III, and αSMA in MCFs (Fig. 2B). Surprisingly, we found that sST2 not only promoted the activation of MCFs but also promoted its proliferation (Fig. 2C). Consequently, we wondered whether sST2 could inhibit MCF senescence. Western blot analysis showed that sST2 significantly inhibited the expression of senescence related proteins p16, p21, and p53 in MCFs (Fig. 2D). Once again, we saw reductions of genes p21 and p53 and increase of gene Sirt1 at the transcript level via RT-PCR (Fig. 2E). These results demonstrate that sST2 can promote the activation of MCFs and inhibit their senescence *in vitro*.

### sST2 Inhibits MCF Senescence through the Sirt1/p53/p21 Signaling Pathway

**Fig. 3:**
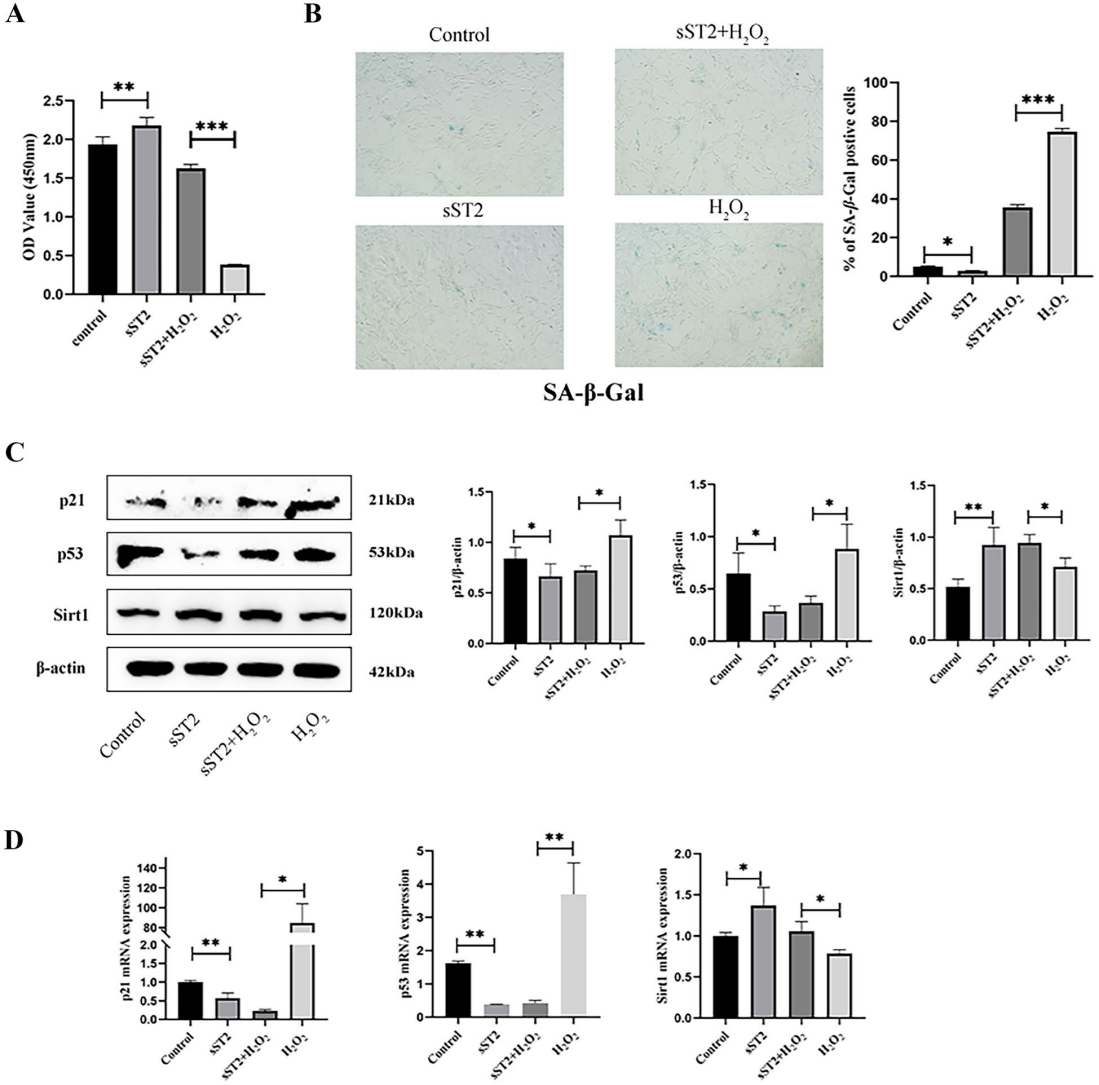
sST2 inhibits MCF senescence through Sirt1/p53/p21 signaling pathway. **(A)** CCK8 assay was used to detect proliferation ability of MCFs*.* **B** SA-β-gal stain was used to detect the senescence of MCFs. **C** Western blot analyses of protein expression of p21, p53, and Sirt1. **D** RT-PCR analyses of the mRNA levels of p21, p53, and Sirt1. Data were represented as the mean ± S.D. Compared with the control group, *p < 0.5; **p < 0.01; ***p < 0.001.

In order to further verify whether sST2 inhibits MCF senescence and determine the specific mechanism whereby it does so, MCFs were divided into four groups: control, sST2, H_2_O_2_, and sST2 + H_2_O_2_. H_2_O_2_, a strong oxidizing agent, was used as a positive control for senescence. A CCK8 test showed that pretreatment with sST2 significantly improved the proliferative capacity of MCFs treated with H_2_O_2_ (Fig. 3A). The numbers of SA-β-gal-positive MCFs were lower in the sST2 + H_2_O_2_ group than those treated with H_2_O_2_ (Fig. 3B), indicating that sST2 pretreatment dramatically inhibited MCF senescence.

Cellular senescence is mainly mediated by the p53/p21 pathway, which, as recently been reported, is regulated by Sirt1 [21]. We therefore detected the expression of p53, p21, and Sirt1 in activated MCFs treated with or without sST2 by RT-PCR and western blot analysis. The results showed that sST2 upregulated the mRNA and protein expression of Sirt1 and downregulated the mRNA and protein expression of senescence related protein p53 and p21 (Fig. 3C, D). Together, these data indicate that sST2 inhibits MCF senescence through the Sirt1/p53/p21 signaling pathway.

### sST2 Promotes MCF Activation by Inhibiting Senescence

**Fig. 4:**
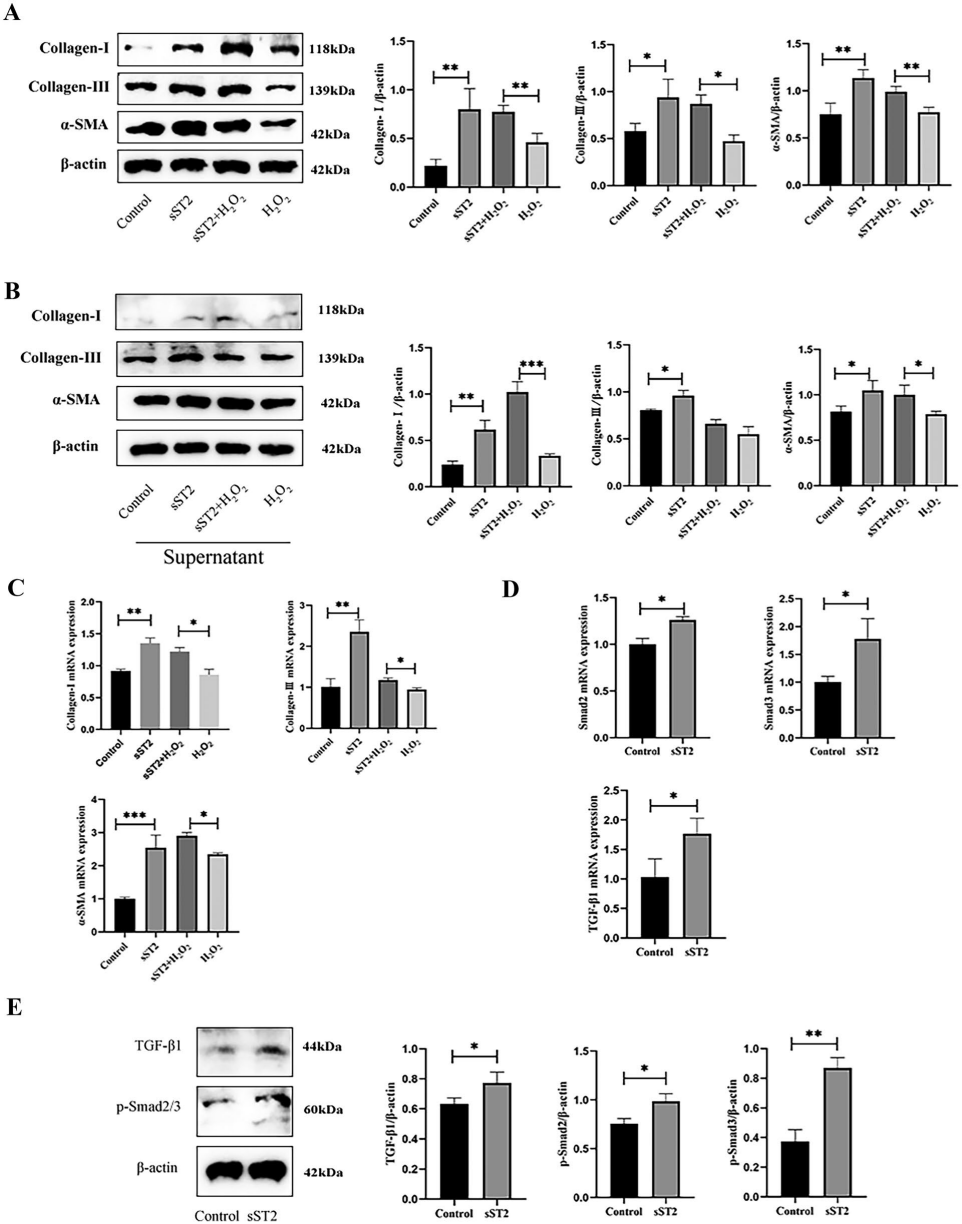
sST2 promotes MCF activation by inhibiting senescence. **A** Western blot analyses of protein expression of Collagen-I, Collagen-III, and α-SMA. **B** Western blot analyses of protein expression of Collagen-I, Collagen-III, and α-SMA. **C** RT-PCR analyses of the mRNA levels of Collagen-I, Collagen-III, and α-SMA. **D** RT-PCR analyses of the mRNA levels of TGF-β1, Smad2, and Smad3. **E** Western blot analyses of protein expression of TGF-β1, p-Smad2, and p-Smad3. Data were represented as the mean ± S.D. Compared with the control group, *p < 0.5; **p < 0.01; ***p < 0.001.

A large number of studies have shown that senescence and fibrosis are closely related. We thus investigated whether sST2 promotes MCF activation by inhibiting senescence. We once again divided MCFs into four groups: control, sST2, sST2 + H_2_O_2_, and H_2_O_2_. The expression of MCF activation-related proteins was detected by western blot analysis. sST2 pretreatment significantly increased the protein levels of Collagen-I, Collagen-III, and αSMA (Fig. 4A).

We next investigated the specific ways in which senescence inhibits MCF activation. Therefore, different treated cell supernatants were used to treat MCFs. In the supernatant of MCFs pretreated with sST2, both the protein and transcript levels of Collagen-I, Collagen-III, and αSMA were significantly increased compared with the supernatant of MCFs only treated with H_2_O_2_. These results suggest that sST2 inhibited MCF senescence and indirectly regulated MCF activation (Fig. 4B, C).

We next sought to understand the specific mechanism leading to MCF activation following sST2 treatment. The TGFβ/Smad pathway is a crucial pathway for fibrosis [22, 23]. We found that sST2 significantly increased the protein and transcript expression of TGFβ1 and pSmad2/3 by western blot analysis and RT-PCR (Fig. 4D, E). Together, these findings suggest that sST2 promotes MCF activation by inhibiting senescence via TGFβ/Smad signaling.

### sST2 Inhibits MCF Senescence and Sirt1/p53/p21 Signaling, Accelerating Cardiac Fibrosis in VMC Mice

**Fig. 5:**
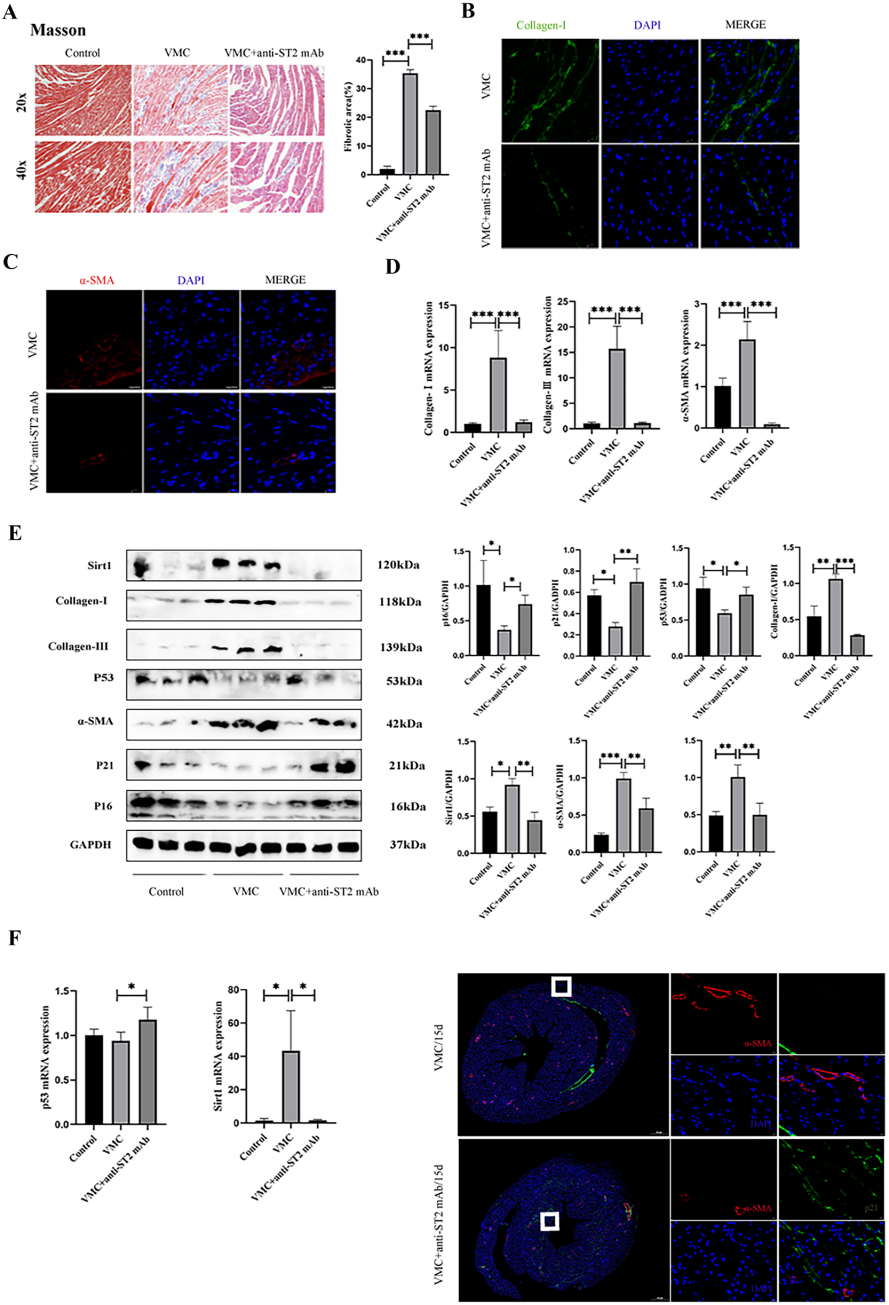
sST2 inhibits MCFs senescence and Sirt1/p53/p21 signaling, accelerating cardiac fibrosis in VMC mice. The heart tissues were collected on day 15 for pathological examination. **A** Representative micrographs of paraffin-embedded heart stained histochemically for Masson’s trichrome (Masson). **B, C** Immunofluorescence images showing the fibroblast activation in heart tissue; *n* = 5 per group. Collagen-I staining is showed in green; α-SMA is shown in red (Scale bar = 20 μm). **D** RT-PCR analysis for Collagen-I/III and α-SMA mRNA in the heart at 15d after VMC. The gene expression level was normalized with that of GAPDH (*n* = 5). **E** Western blotting for Sirt1, Collagen-I/III, p53, α-SMA, p21, and p16. GAPDH was used as the loading control. Protein expression relative to β-actin was assessed by densitometric analysis. **F** RT-PCR analysis for Sirt1/p53 mRNA in the heart at 15d after VMC. The gene expression level was normalized with that of GAPDH (*n* = 5). **G** Representative micrographs of cell immunofluorescently stained for p21 and α-SMA, with DAPI staining the nucleus. Data were represented as the mean ± S.D. Compared with the control group, *p < 0.5; **p < 0.01; ***p < 0.001.

Finally, we investigated sST2 was directly responsible for promoting fibrosis in MCFs. To this end, anti-ST2 mAb was injected intraperitoneally into VMC mice. The myocardium of VMC mice displayed characteristics typical of fibrosis, as evidenced by increased interstitial ECM deposition visualized by Masson staining analysis. However, this was not observed in the anti-ST2 mAb group (Fig. 5A). Immunofluorescent staining of Collagen-I and αSMA showed a decrease in VMC mice treated with anti-ST2 compared to untreated VMC mice (Fig. 5B, C). Parallel results were observed at the transcript level (Fig. 5D). In addition, western blot analysis showed that sST2 decreased the expression of the senescence-related genes p53 and p21 and increased Sirt1 in cardiac tissue of VMC mice. Fibrosis-related genes Collagen-I/III and αSMA were significantly decreased in VMC mice treated with sST2 neutralizing antibody (Fig. 5E, F). To further confirm the effect of MCF senescence on cardiac fibrosis, we costained the senescence marker p21 with the specific MCF activation marker αSMA. We found that, in the anti-ST2mAb group, p21, but not αSMA, was highly expressed. However, p21 was significantly reduced in the VMC group (Fig. 5G). These results suggested that sST2 accelerated cardiac fibrosis by inhibiting cellular senescence and Sirt1/p53/p21 signaling and promoting collagen deposition.

## DISCUSSION

The main purpose of this paper was to investigate the effect of sST2 on VMC and to elucidate the relevant mechanism. Following VMC, the level of sST2 increased significantly, and cardiac fibrosis also appeared [24]. Our study shows that sST2 can induce MCF activation and collagen secretion, leading to cardiac fibrosis. Notably, sST2 promotes cardiac fibrosis by inhibiting MCF senescence. Therefore, the present study lays out a mechanism whereby sST2 activates MCFs during VMC by impeding senescence in these cells.

Most previous studies have emphasized that sST2 plays a harmful role as a soluble decoy receptor by blocking the function of IL-33, which has anti-inflammatory and antioxidant functions [8, 25, 26]. In fact, sST2 can make a difference independently of IL-33. Previous studies have shown that sST2 can promote cardiac fibrosis and increase inflammatory molecules production through the production of reactive oxygen species [11]. In the present study, we expand upon previous findings by showing that sST2 levels are elevated in VMC, which can promote cardiac fibrosis by inhibiting MCF senescence. Our *in vivo* experiments demonstrate that sST2 can increase collagen deposition in the heart, increase inflammation, and reduce MCF senescence. We also demonstrate *in vitro* that the stimulation of sST2 promotes MCF activation through TGFβ/Smad2/3 signaling and inhibits cellular senescence by Sirt1/p53/p21 signaling. Our study further strengthens the idea of sST2 as a predictor of cardiomyopathy and suggests that sST2 can be a therapeutic target for cardiac fibrosis.

Many signaling pathways are involved in the regulation of cellular senescence. p53 and its downstream signaling factor p21 play a crucial role in the regulation of senescence [27–29]. Overexpression of p53 and p21 can increase SA-β-gal activity and induce cell cycle arrest [30]. In this study, we found that sST2 upregulated the expression of Sirt1 and inhibited the expression of p53 and p21. In addition, we verified by β-galactosidase staining and CCK8 assay that sST2 inhibited MCF senescence by sST2.

**Fig. 6:**
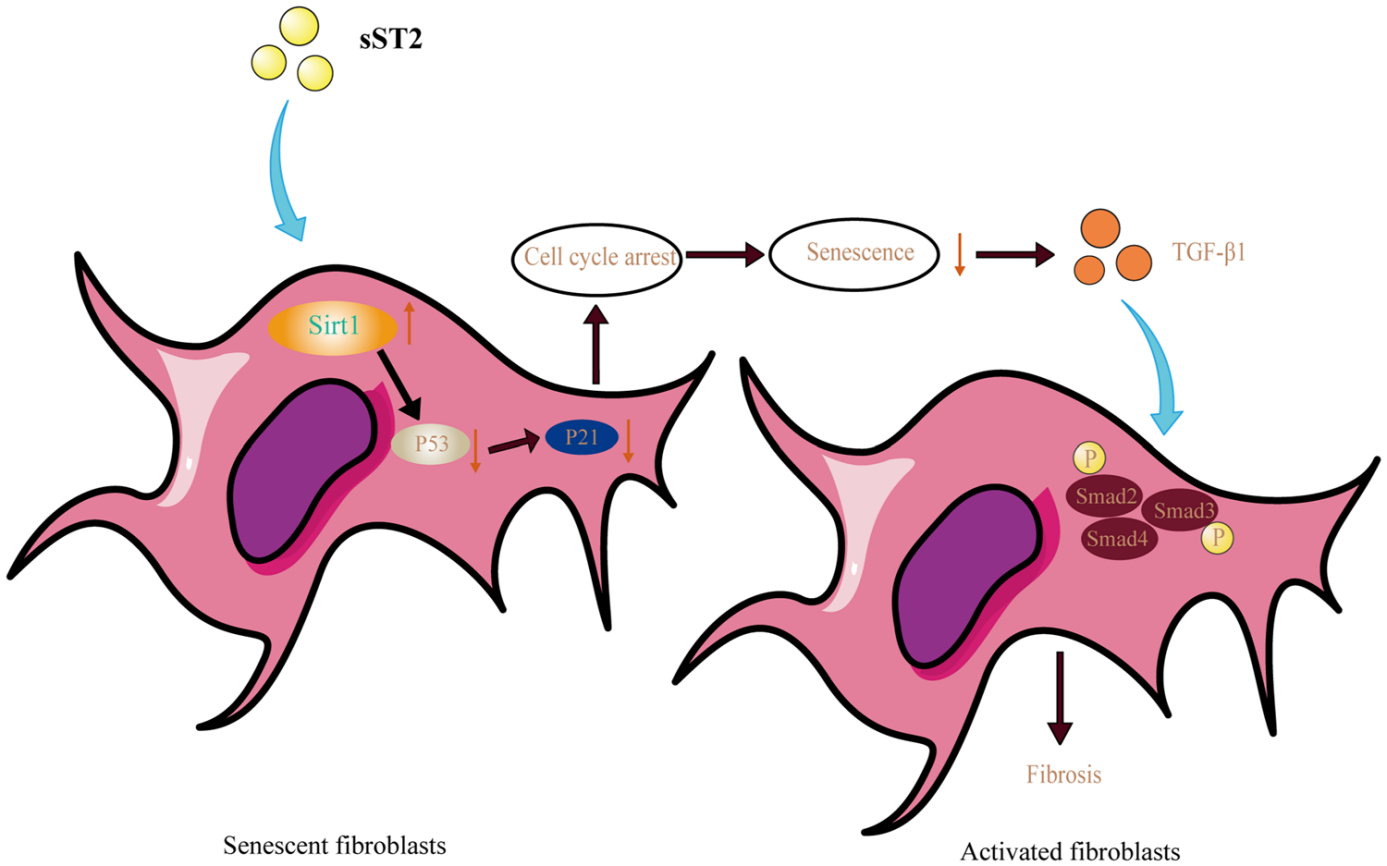
Elevated sST2 in the VMC could have a deleterious effect on the heart by inhibiting senescence of MCF, leading to cardiac fibrosis.

TGFβ/Smad2/3 is an important signaling pathway in cardiac fibrosis [31–33]. TGFβ, as a cytokine, promotes MCF activation and extracellular matrix production and plays a key role in cardiac fibrosis [34]. The TGFβ receptor is a heterodimeric receptor complex composed of TGFβ type I and II receptors [23]. It can phosphorylate Smad2 and 3 transcription factors and make them transfer the signal to the nucleus [35–37]. In this paper, we found that sST2 stimulation led to MCF activation and significantly increased expression of TGFβ1, pSmad2, and pSmad3. Furthermore, we also found that sST2 can activate MCFs by inhibiting MCF senescence and that this effect can be achieved via a paracrine manner, as evidenced by our observation that culture supernatant of MCFs treated with sST2 and H_2_O_2_ could promote the activation of other MCFs, while the culture supernatant of senescent MCFs treated with hydrogen peroxide alone could not (Fig. 6).

In summary, the present study shows that elevated sST2 in VMC can have a deleterious effect on the heart by inhibiting senescence of MCF, leading to cardiac fibrosis. Therefore, our findings may provide a novel therapeutic target to alleviate cardiac fibrosis.

## Data Availability

The data used or analyzed during the current study are available from the corresponding author on reasonable request.
